# Combined Endobronchial and Transesophageal Approach of an Ultrasound Bronchoscope for Mediastinal Staging of Lung Cancer

**DOI:** 10.1371/journal.pone.0091893

**Published:** 2014-03-14

**Authors:** Kyung Jong Lee, Gee Young Suh, Man Pyo Chung, Hojoong Kim, O. Jung Kwon, Joungho Han, Sang-Won Um

**Affiliations:** 1 Division of Pulmonary and Critical Care Medicine, Department of Medicine, Samsung Medical Center, Sungkyunkwan University School of Medicine, Seoul, Korea; 2 Department of Pathology, Samsung Medical Center, Sungkyunkwan University School of Medicine, Seoul, Korea; University of North Dakota, United States of America

## Abstract

**Background:**

We evaluated the utility of a combined approach using endobronchial ultrasound-guided transbronchial needle aspiration (EBUS-TBNA) and transesophageal bronchoscopic ultrasound-guided fine-needle aspiration (EUS-FNA-B/E) for mediastinal staging of lung cancer.

**Methods:**

An EBUS-TBNA database was analyzed retrospectively. EUS-FNA-B/E was performed after EBUS-TBNA when mediastinal lymph nodes were not accessible using EBUS-TBNA or when tissue sampling using EBUS-TBNA alone was inadequate.

**Results:**

During the study period, 44 patients were enrolled. EBUS-TBNA and EUS-FNA-B/E were performed on 79 and 52 lymph nodes, respectively. The sensitivity, specificity, and accuracy of mediastinal N-staging using EBUS-TBNA alone were 79%, 100%, and 84%, respectively. The sensitivity, specificity, and accuracy of mediastinal N-staging using a combination of EBUS-TBNA and EUS-FNA-B/E were 100%, 100%, and 100%, respectively. Significant differences in sensitivity (*P* = 0.008) and accuracy (*P* = 0.004) of mediastinal N-staging were evident when EBUS-TBNA alone and the combined procedure were compared. The nodal stage shifted higher after use of the EUS-FNA-B/E procedure in six cases (13%). No serious complication associated with the procedures was noted.

**Conclusions:**

Use of a combination of EBUS-TBNA and EUS-FNA-B/E can afford better sensitivity and accuracy of mediastinal N-staging compared with use of EBUS-TBNA alone. Such combined procedures should be considered for examination of lesions that are inaccessible or difficult to access by EBUS-TBNA.

## Introduction

Lung cancer is the leading cause of mortality associated with malignancy despite recent advances in lung cancer management strategies [1]. The possibility of resection is one of the most significant factors influencing the treatment of lung neoplasms if no obvious distant metastasis is evident.

Thus, mediastinal lymph node assessment is important when assessing the resectability of lung cancer in the absence of extra- or intra-thoracic metastasis [2], [3]. Mediastinoscopy plays a key role in staging of mediastinal lymph nodes suspicious for metastasis on positron-emission-tomography imaging, although systemic lymph node dissection requires the use of general anesthesia [4].

Endobronchial ultrasound-guided transbronchial needle aspiration (EBUS-TBNA) has been developed to diagnose and stage non-small cell lung cancer; the technique is an alternative to mediastinoscopy [5]. EBUS-TBNA cannot access all mediastinal lymph nodes. A combination of endoscopic ultrasound-guided fine-needle aspiration (EUS-FNA) and EBUS-TBNA can afford a more accurate and systematic assessment of the mediastinum [6]. However, use of this combination is associated with several limitations when mediastinal node staging is performed in clinical practice. The procedure requires expert endoscopists and expensive equipment, increasing medical costs and the time required for lung cancer evaluation [7]. It would be better if EBUS-TBNA and EUS-FNA could be performed sequentially, in the same setting, by the same operator.

The same operator performed both EBUS-TBNA and transesophageal bronchoscopic ultrasound-guided fine needle aspiration (EUS-FNA-B/E), using an EBUS bronchoscope, in a bronchoscopy suite, when mediastinal lymph nodes could not be accessed using EBUS-TBNA or the tissue samples obtained by EBUS-TBNA alone were inadequate. In the present study, we compared the diagnostic performances of mediastinal N staging by EBUS-TBNA alone with that afforded by a combination of EBUS-TBNA and EUS-FNA-B/E.

## Materials and Methods

### Study Subjects

Data collected between May 2010 and February 2012 at the Samsung Medical Center (Seoul, South Korea) were respectively analyzed. A total of 605 patients underwent EBUS-TBNA for staging and diagnosis of primary lung cancer, extrapulmonary malignancies, lymphoma, tuberculous lymphadenitis, and sarcoidosis. The study was approved by the Institutional Review Board of the Samsung Medical Center. The requirement for informed consent of the individual patients was waived given the retrospective nature of the study by the Institutional Review Board of the Samsung Medical Center. Patient information was anonymized and de-identified prior to analysis. Patients who underwent EBUS-TBNA followed by EUS-FNA-B/E to diagnose and stage confirmed or suspicious lung cancer were included. Prior to each procedure, PET/CT was routinely used for assessment of mediastinal lymph nodes and other metastatic sites. Patients exhibiting metastasis to extrathoracic sites were excluded. Patients underwent EUS-FNA-B/E after EBUS-TBNA when mediastinal lymph nodes were not accessible by EBUS-TBNA or when adequate tissue samples could not be obtained using EBUS-TBNA alone.

### EBUS-TBNA

EBUS-TBNA was performed in a bronchoscopy suite by two bronchoscopists (KJ Lee and SW Um). A routine endobronchial inspection was initially performed. A convex-probe ultrasound bronchoscope fitted with a linear transducer (7.5 MHz) was used in both EBUS-TBNA and EUS-FNA-B/E (CP-EBUS, BF-UC206F-OL8, Olympus, Tokyo, Japan). Lymph node images were also processed using an ultrasonic scanner (EU-C2000; Olympus, Tokyo, Japan). Conscious sedation with midazolam was applied during the EBUS-EBNA procedure, which was performed on an inpatient basis. Nebulized lidocaine (4%) was used to achieve local anesthesia prior to each procedure. Bolus doses of 1.3% lidocaine were administered during the procedure, as needed. The use of EBUS-TBNA to assess mediastinal lymph nodes was indicated when nodes more than 10 mm in diameter (short axis diameter) on chest CT or when increased mediastinal lymph node FDG uptake (compared to that of surrounding tissue) was evident. A 22-gauge needle (NA-201SX-4022; Olympus) was used to obtain TBNA biopsy material. Lymph node station was defined in accordance with the international staging system of the International Association for the Study of Lung Cancer (IASLC) [8]. All aspirate specimens were smeared and immediately fixed on glass slides and sent to a pathologist for cytological and/or histological examination. We did not implement Rapid On-Site Cytopathological Evaluation (ROSE).

### Transesophageal bronchoscopic ultrasound-guided fine-needle aspiration (EUS-FNA-B/E) using a EBUS bronchoscope

After completion of EBUS-guided TBNA, the EBUS bronchoscope was re-inserted through the esophagus from the pharynx. To facilitate insertion of the bronchoscope and visualization of the esophagus, oxygen (1–2 l/min) was delivered through a working channel [9]. The scope was inserted gently, and the aortic arch, descending aorta, left main pulmonary artery, and heart were searched for landmarks. A 22-gauge needle was used to sample lymph nodes through the esophageal wall. All aspirate specimens were smeared and fixed immediately on glass slides and sent to a pathologist for cytological and/or histological examination.

### Definitions of diagnostic standards

Final nodal stage was defined based on evaluation of the data from all of EBUS-TBNA, EUS-FNA-B/E, and surgery. Metastasis was defined by pathological confirmation via EBUS-TBNA, EUS-FNA-B/E, mediastinoscopy, or mediastinal lymph node dissection. Benign lymph nodes were confirmed by surgery to treat suspected lesions. Patients who had yielded benign results on EBUS-TBNA and EUS-FNA-B/E but whose benign status was not confirmed surgically were excluded from diagnostic performance analyses.

### Statistical analysis

Mediastinal N-staging results from PET-CT, EBUS-TBNA, and the combination of EBUS-TBNA and EUS-FNA-B/E, were analyzed. Comparisons of mediastinal staging afforded by use of EBUS-TBNA alone and the combined procedure were made using McNemar’s test and the Bennett method [10]. Comparisons of mediastinal N-staging afforded by PET/CT and the combination of EBUS-TBNA and EUS-FNA-B/E were also made. The sensitivity, specificity, positive predictive value (PPV), negative predictive value (NPV), and accuracy of mediastinal N staging were analyzed on a per-person basis using standard definitions. All tests were two-tailed and statistical significance was defined when the comparative *P* value was <0.05. The SPSS software (version 20.0; SPSS Inc., Chicago, IL,) was used for statistical analysis.

## Results

### Study patients

We included 44 patients. Of these, 82% were males and the median age was 66 years (range, 43–86) years. The median procedure time from the start of EBUS-TBNA to the end of EUS-FNA-B/E was 40 min (range, 15–70 min). No serious complication was associated with use of the EBUS-TBNA or EUS-FNA-B/E procedures. Baseline characteristics of all patients are shown in Table 1.

**Table 1:** Demographic characteristics of the patients (n = 44).

Characteristics	Number (%) or median (range)
**Age, years**	66.0 (43–86)
**Male/female (%)**	36 (81.8)/ 8 (18.2)
**Histological type**	
Adenocarcinoma	19 (43.2)
Squamous cell carcinoma	20 (45.5)
Small cell carcinoma	3 (6.8)
NSCLC,NOS	2 (4.5)
**Nodal size, mm**	
Short axis	10.0 (4–58)
Long axis	14.0 (6–72)
**Procedure time, min**	40 (15–70)
**Aspiration per lymph node**	2 (1–4)

NSCLC, non-small-cell lung cancer; NOS; not otherwise specified.

### Lymph nodes examined

Totals of 79 and 52 lymph nodes were examined using EBUS-TBNA and EUS-FNA-B/E, respectively (Table 2). The median number of aspirations per lymph node was 2 (range, 1–4). The most frequently aspirated nodes by in EBUS-TBNA were the subcarinal lymph nodes (7) while the left paratracheal lymph nodes (in the 4L region) were most often accessed using EUS-FNA-B/E (Table 2). Both EBUS-TBNA and EUS-FNA-B/E were performed at 19 nodal stations (2 at station 1R, 11 at station 4L and 6 at station 7). Among the 79 lymph nodes examined by EBUS-TBNA, 24, 53, and 2 yielded malignant, benign, and inadequate results by cytology and 26, 52, and 1 malignant, benign, and inadequate results by histology, respectively. Of the 52 lymph nodes examined by EUS-FNA-B/E, 21, 30, and 1 yielded malignant, benign, and inadequate results by cytology and 24 and 28 had malignant and benign by histology.

**Table 2:** Mediastinal lymph nodes examined by endobronchial ultrasound-guided transbronchial needle aspiration (EBUS-TBNA) and transesophageal bronchoscopic ultrasound-guided fine-needle aspiration (EUS-FNA-B/E).

Nodal stations	Number of lymph nodes	
	EBUS-TBNA (n = 79)	EUS-FNA-B/E (n = 52)
**1R**	2	4
**2R**	8	1
**3P**	0	2
**4R**	21	0
**4L**	18	24
**5**	0	7
**7**	23	6
**8**	0	6
**10L**	0	2
**11R**	2	0
**11L**	5	0

EBUS-TBNA: endobronchial ultrasound-guided transbronchial needle aspiration; EUS-FNA-B/E: transesophageal bronchoscopic ultrasound-guided fine-needle aspiration.

### Final nodal stage

**Figure 1:**
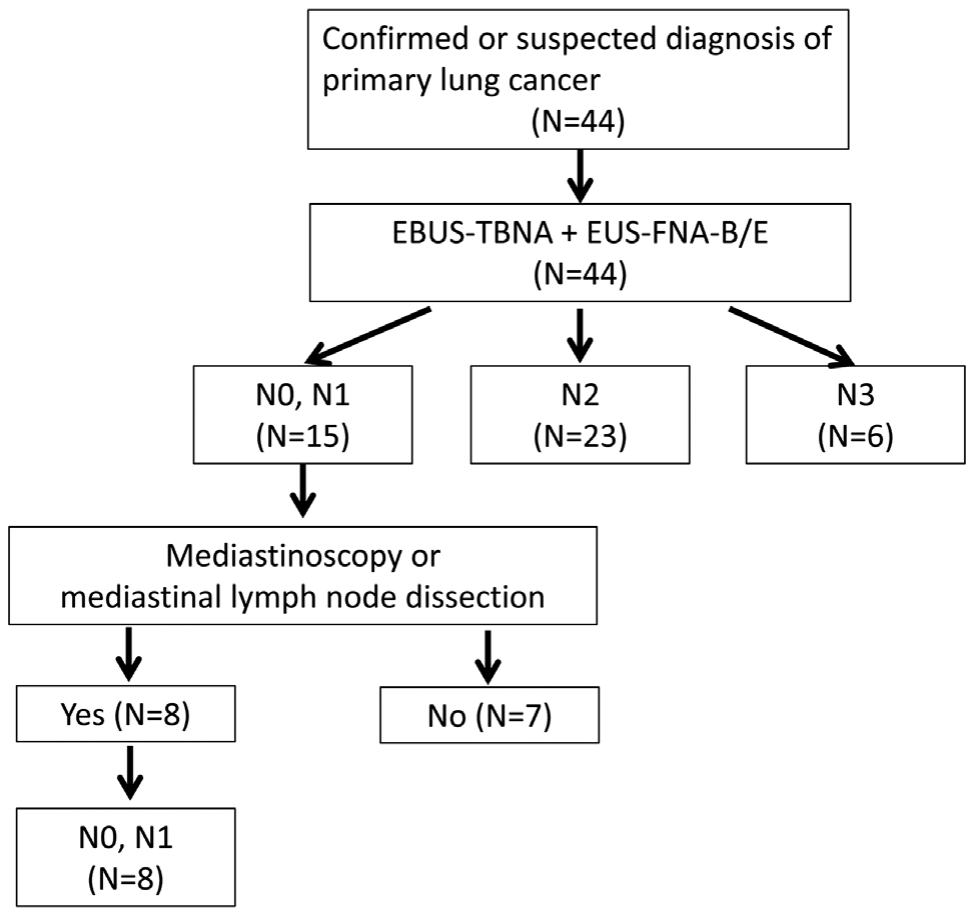
Diagnostic algorithm of study patients. EBUS-TBNA, endobronchial ultrasound-guided transbronchial needle aspiration; EUS-FNA-B/E, transesophageal bronchoscopic ultrasound-guided fine-needle aspiration.

The final stages of study patients are summarized in Figure 1. Six and 23 patients were confirmed as having N3 and N2 disease after combined EBUS-TBNA and EUS-FNA-B/E, respectively. Of 15 patients who yielded benign results after the combined procedure, 8 underwent mediastinoscopy (n = 4) or mediastinal lymph node dissection (n = 4); these patients were confirmed to have N0 or N1 disease. Seven patients who did not undergo surgical confirmation of benign status were excluded from diagnostic performance analyses.

### Mediastinal N-staging analyses

The sensitivity, specificity, PPV, NPV, and accuracy of mediastinal N-staging using PET/CT, EBUS-TBNA, and combined EBUS-TBNA and EUS-FNA-B/E data are summarized in Table 3. The sensitivity of mediastinal N-staging by PET-CT was 93%, the specificity 37%, the PPV 84%, the NPV 60%, and the accuracy was 81%. Use of EBUS-TBNA alone for mediastinal N-staging yielded a sensitivity of 79%, a specificity of 100%, a PPV of 100%, an NPV of 57%, and an accuracy of 84%. The combined procedure yielded a sensitivity of 100%, a specificity of 100%, a PPV of 100%, an NPV of 100%, and an accuracy of 100%. Significant differences in mediastinal N-staging sensitivity (*p* = 0.008) and accuracy (*P* = 0.004) were evident when EBUS-TBNA alone and the combined procedure were compared. Differences occurred in the diagnostic specificity (*P* = 0.025) and PPV (*P* = 0.039) between PET/CT and the combined procedure. Six patients (13.6%) were up-shifted to higher nodal stages after EUS-FNA-B/E data were added to EBUS-TBNA information. Nodal stage changes are summarized in Table 4. EUS-FNA-B/E could detect nodal metastasis in stations 1R, 4L, 5, 7, and 8.

**Table 3:** Diagnostic performances of PET/CT, EBUS-TBNA, and the combination of EBUS-TBNA and EUS-FNA-B/E for the detection of mediastinal metastasis on a per person basis.

	Sensitivity	Specificity	PPV	NPV	Accuracy
**PET-CT**	93.1(27/29)	37.5 (3/8)	84.3 (27/32)	60.0 (3/5)	81.0 (30/37)
**EBUS**	79.3 (23/29)	100 (8/8)	100 (23/23)	57.1 (8/14)	83.7 (31/37)
**EBUS+EUS**	100 (29/29)	100 (8/8)	100 (29/29)	100 (8/8)	100 (37/37)
**p-value** *	0.083	0.025	0.039	0.205	0.004
**p-value** **	0.008	NA	NA	0.053	0.004

Data are presented as n (%).

* p-values from the comparisons of PET-CT and EBUS+EUS-FNA-B/E.

** p-values from the comparisons of EBUS-TBNA and EBUS+EUS-FNA-B/E.

PPV: positive predictive value; NPV: negative predictive value; PET-CT: positron-emission tomography with computed tomography; EBUS-TBNA: endobronchial ultrasound-guided transbronchial needle aspiration; EUS-FNA-B/E: transesophageal bronchoscopic ultrasound-guided fine-needle aspiration; NA: not applicable.

**Table 4:** The six cases of increased mediastinal stage after addition of EUS-FNA-B/E.

Case	Gender	Age	EBUS-TBNA	EUS-FNA-B/E	Shift of N stage
1	M	77	7: benign	7:adenocarcinoma	N0 → N2
2	M	70	4R: benign	5: adenocarcinoma	N0 → N2
			7: benign		
			4L: benign		
3	M	69	4R:Squamous cell carcinoma	4L: Squamous cell carcinoma	N2 → N3
4	M	57	2R: benign	5: adenocarcinoma	N0 → N2
			4L: benign		
5	M	61	7: benign	1R: adenocarcinoma	N0 → N3
			8: benign		
6	M	72	11R: benign	8: squamous cell carcinoma	N0 → N2
			7: benign		
			4R: benign		
			4L: benign		

EBUS-TBNA: endobronchial ultrasound-guided transbronchial needle aspiration; EUS-FNA-B/E: transesophageal bronchoscopic ultrasound-guided fine-needle aspiration.

## Discussion

In the present study, the sensitivity of mediastinal N-staging afforded by combined EBUS-TBNA and EUS-FNA-B/E was significantly higher than that of EBUS-TBNA alone. Upstaging was noted in six of 44 patients (13.6%) by adding EUS-FNA-B/E. Five of these six patients were re-staged from N0 to N2 or N3, and one patient was re-staged from N2 to N3.Mediastinal lymph node evaluation permitting exact staging is important in predicting prognosis and planning treatment strategies for lung cancer [11]. FDG-PET assessment of mediastinal lymph nodes yielded a sensitivity of 73% and a specificity of 92% [12]. Despite this excellent specificity of integrated PET-CT, these data suggest that tissue samples should be obtained to confirm mediastinal node staging. Currently, video-assisted mediastinoscopy is the gold standard for evaluation of all accessible mediastinal lymph nodes in lung cancer patients. A review of video-assisted mediastinoscopic lymphadenectomy in patients with NSCLC reported a sensitivity of 90% and a false-negative rate of 7%. Moreover, the procedure-related morbidity and mortality rates of mediastinoscopic lymphadenectomy are 2% and 0.08%, respectively [3].

EBUS-TBNA is a non-invasive procedure used to aspirate mediastinal nodes employing an ultrasonic probe adopted for use in bronchoscopy. The procedure has several advantages, including a lower risk of morbidity and mortality and an increase in accessible regions, including the hilar and interlobar lymph nodes, compared with mediastinoscopy [13]. A meta-analysis performed by Gu *et al*. showed that use of the technique afforded a sensitivity of 93% and a specificity of 100% [14]. EBUS-TBNA does not allow access to the paraaortic or paraesophageal lymph nodes via a transbronchial approach. The technique also cannot be used to access the deep left lower paratracheal station because of the limited angulation afforded by bronchoscopy [15].

To overcome these limitations, a combined procedure including EUS-FNA was developed. The use of EUS-FNA with EBUS-TBNA enabled accurate and systematic assessment of mediastinal lymph nodes [16]. This combined approach was associated with a significantly higher sensitivity and specificity for non-small cell lung cancer staging than were EBUS-TBNA or EUS-FNA alone [17]. EUS-FNA can access additional lymph node stations that are inaccessible using an endobronchial approach. The systematic approach has a sensitivity of 68–96% and an NPV of 75–97% [17]–[19]. The results suggest that use of the combined procedure enables more accurate mediastinal staging.

A transesophageal approach using a single EBUS bronchoscope was performed in the same bronchoscopy suite for more accurate evaluation of mediastinal staging in this study. In total, 112 lymph nodes were sampled in 44 patients by a single ultrasound bronchoscope through the bronchus and esophagus. We sampled the same lymph node using EUS-FNA-B/E if EBUS-TBNA was unable to acquire sufficient tissue. This could occur because the lymph node was located in a deep station that was not readily visible using the ultrasound probe or because there was insufficient tissue from aspiration due to poor alignment. Only 71% of all sampled lymph nodes were accessible and sampled by EBUS-TBNA alone, but the proportion of accessible lymph nodes increased with the addition of the transbronchial approach using a single ultrasound bronchoscopy. In this study, 19 nodal stations (2 at station 1R, 11 at station 4L, and 6 at station 7) were examined by both EBUS-TBNA and EUS-FNA-B/E at the bronchoscopist’s discretion, as it seemed that sufficient tissue was not obtained by EBUS-TBNA alone. Material aspirated from three nodal stations using EBUS-TBNA and EUS-FNA-B/E yielded different results. These three nodal stations were diagnosed as metastatic by EUS-FNA-B/E despite being negative on EBUS-TBNA. The diagnostic sensitivities of EBUS-TBNA and EUS-FNA-B/E were 75% (9/12) and 100% (12/12) for these 19 nodal stations, respectively.

The advantages of the combined procedure using a single ultrasonic bronchoscope have been reported in two studies. Bo *et al*. evaluated the use of combined EBUS-B-FNA and EBUS-TBNA in 150 patients and claimed a 97% diagnostic accuracy for detection of mediastinal metastasis [20]. Herth *et al*. also used a transbronchial approach followed by transesophageal needle aspiration employing an ultrasonic bronchoscope and reported a 96% sensitivity of cancer detection and a 96% NPV on examination of 619 nodes from 139 patients with suspected lung cancer [21]. We also found that the accuracy of mediastinal staging was improved after addition of EUS-FNA-B/E in clinical practice. The combined procedure yielded more accurate results than those afforded by PET-CT or EBUS-TBNA alone.

Use of EUS-FNA overcomes the limitations of EBUS-TBNA for mediastinal nodal staging of lung cancer. However, the combination of EBUS-TBNA and EUS-FNA increases the medical cost and delays the diagnosis, as EUS-FNA would need to be performed by expert endoscopists during a different session. The combination of EBUS-TBNA and EUS-FNA-B/E using a single ultrasonic bronchoscope is a useful alternative method because EUS-FNA-B/E by a bronchoscopist does not increase the medical cost and it could be performed immediately after EBUS-TBNA. The median EBUS-TBNA procedure time was 20 min at our institution, and that of the combined EBUS-TBNA and EUS-FNA was 40 min. Therefore, the combined procedure increased the procedure time by only 20 min compared with EBUS-TBNA alone.

Our study had several limitations. First, all participants were recruited retrospectively and our sample size was small. Thus, The possibility of selection bias exists; we may have overestimated the utility of the combined procedure. Therefore, our data should be interpreted conservatively as only 44 (7.3%) of the 605 patients were enrolled and 13% upstaging in 44 patients represents only 1% of 605 patients. Second, of 15 patients who had negative N2 or N3 nodes after EBUS-TBNA and EUS-FNA-B/E, only 8 underwent surgical confirmation using mediastinoscopy or mediastinal lymph node dissection. This may contribute to overestimation of the diagnostic performance of the combined procedure. If the seven patients who did not undergo surgical confirmation are considered to be false-negatives, the sensitivity, NPV, and accuracy of mediastinal N-staging by EBUS-TBNA and the combined procedure would be 64% (23/36), 38% (8/21), and 70% (31/44); and 81% (29/36), 53% (8/15), and 84% (37/44), respectively. However, significant differences would remain in terms of both the sensitivity (*P* = 0.016) and accuracy (*P* = 0.008) of mediastinal N-staging conducted using the EBUS-TBNA and combined procedure.

In conclusion, use of a combination of EBUS-TBNA and EUS-FNA-B/E afforded greater diagnostic sensitivity and accuracy of mediastinal N-staging than did EBUS-TBNA alone. The combined procedure can be used for more accurate mediastinal staging of lesions difficult to access using EBUS-TBNA only.
